# Increasing Hepatitis C treatment uptake among HIV-infected patients using an HIV primary care model

**DOI:** 10.1186/1742-6405-10-9

**Published:** 2013-03-28

**Authors:** Edward R Cachay, Lucas Hill, Craig Ballard, Bradford Colwell, Francesca Torriani, David Wyles, William C Mathews

**Affiliations:** 1 Department of Medicine, Owen Clinic, University of California at San Diego, 200 W. Arbor Drive, San Diego, CA, 92103-8681, USA; 2 Skaggs School of Pharmacy and Pharmaceutical Sciences, University of California at San Diego, San Diego, CA, USA; 3 Department of Medicine, Division of Infectious Diseases, University of California at San Diego, San Diego, CA, USA

**Keywords:** HIV, HCV treatment, Primary care, Hepatology

## Abstract

**Background:**

Access to Hepatitis C (HCV) care is low among HIV-infected individuals, highlighting the need for new models to deliver care for this population.

**Methods:**

Retrospective cohort analysis that compared the number of HIV patients who initiated HCV therapy: hepatology (2005–2008) vs. HIV primary care model (2008–2011). Logistic-regression modeling was used to ascertain factors associated with HCV therapy initiation and achievement of sustained viral response (SVR).

**Results:**

Of 196 and 163 patients that were enrolled in the HIV primary care and hepatology models, 48 and 26 were treated for HCV, respectively (p = 0.043). The HIV/HCV-patient referral rate did not differ during the two study periods (0.10 vs. 0.12/patient-yr, p = 0.18). In unadjusted analysis, predictors (p < 0.05) of HCV treatment initiation included referral to the HIV primary care model (OR: 1.7), a CD4+ count ≥400/mm3 (OR: 1.8) and alanine aminotranferase level ≥63U/L (OR: 1.9). Prior psychiatric medication use correlated negatively with HCV treatment initiation (OR: 0.6, p = 0.045). In adjusted analysis the strongest predictor of HCV treatment initiation was CD4+ count (≥400/mm3, OR: 2.1, p = 0.01). There was no significant difference in either clinic model (primary care vs. hepatology) in the rates of treatment discontinuation due to adverse events (29% vs. 16%), loss to follow-up (8 vs. 8%), or HCV SVR (44 vs. 35%).

**Conclusions:**

Using a HIV primary care model increased the number of HIV patients who initiate HCV therapy with comparable outcomes to a hepatology model.

## Introduction

Access to hepatitis C (HCV) treatment remains low in HIV-infected individuals in the USA [1-3], resulting in increased morbidity and cost due to HCV related complications [4,5].

The following factors account in part for the low treatment rates of HCV in the HIV population: increased prevalence of competing medical co-morbidities [6], ongoing substance/alcohol dependence and neuropsychiatric disease [7]. Additional disincentives for patients to seek HCV treatment include: low efficacy and severe side effects associated with conventional HCV therapies (pegylated interferon and ribavirin) [8]; long waiting time prior to HCV intake appointment in sub-specialty clinics [9]; and commuting to a different location from where they routinely receive their HIV care [10,11].

In April 2008, the University of California at San Diego (UCSD) transitioned from a hepatology specialty model to an HIV primary care hepatitis program for the management of HCV in HIV-infected patients without advanced cirrhosis. Many centers around the country with a high burden of HIV/HCV co-infection have implemented similar multidisciplinary co-infection programs, but there is little data describing the structure, processes, and outcomes of these programs. Thus, we sought to compare the rates of referral, treatment initiation and completion between a prior hepatology-managed co-infection clinic and a subsequent HIV primary care managed co-infection clinic after the first three years of transition.

## Methods

### Study design and patients

We compared two adult cohorts: (1) UCSD hepatology specialty model (1 Jan 2005–31 March 2008) and (2) the UCSD Owen hepatitis co-infection clinic (1 April 2008–30 July 2011). Inclusion criteria required: (1) a documented clinical decision regarding HCV treatment eligibility; (2) if treated, HCV treatment consisting of pegylated interferon and ribavirin. The study was approved by the UCSD Human Research Protection Program.

### Description of HIV primary care model

The UCSD Owen clinic cares for more than 3,000 HIV-infected patients of whom approximately 20% are co-infected with HCV. Prior to April 2008 a team of hepatologists managed a once weekly clinic for assessment of HIV/HCV patients. There were no explicit inclusion criteria for referral to the traditional hepatology-based clinic. Historically, HIV providers made referral decisions based on their own referral criteria. The new hepatitis co-infection primary care model also operates as one clinic session per week and is staffed by three HIV clinicians with Infectious Diseases certification, a psychiatrist, two clinical pharmacists specialized in HIV care, one health educator and a substance counselor. There was no change in procedures for patient referral to the primary care managed co-infection clinic nor were there any other structural changes to the HIV clinic during the transition process; all HIV patients under care with viral hepatitis co-infection could be referred by their HIV primary care provider for evaluation. Every patient underwent a detailed clinical, laboratory and imaging (ultrasound and/or abdomino-pelvic computer tomography) assessment for staging degree of liver disease according to standard of care and ruling out indirect evidence of advanced liver disease and/or portal hypertension [12]. Patients with HCV genotype 1 or 4 were offered a liver biopsy performed by the interventional radiology service unless patients declined the procedure. Esophagogastroduodenoscopies when required were completed by the gastroenterology service. Our minimal requirements for HCV treatment eligibility were: (1) undetectable HIV viral load and CD4 cell count ≥ 200/cm^3^ if on antiretroviral therapy; (2) CD4 cell count ≥ 500/cm^3^ irrespective of HIV viral load value if naïve to antiretroviral therapy; (3) absence of Child B or C liver cirrhosis; (4) stable concurrent medical co-morbidity; (5) favorable recommendation from the team’s psychiatrist; (6) registration in the San Diego needle exchange program in the case of ongoing parenteral drug use with documentation of controlled HIV infection as in (1) and (2), plus documentation of no missed clinic appointments during HCV staging process; and (7) alcohol sobriety for at least 3 months prior to HCV treatment initiation. Homeless patients were required to receive all weekly pegylated interferon injections in clinic.

### HCV treatment monitoring strategies and role of pharmacists in the HIV primary care model

Following favorable consideration to initiate HCV therapy, a patient was assigned to one of three groups for HCV treatment monitoring: Group 1 had patients without major significant medical co-morbidity, social barriers and no on-going illicit substance use. Group 2 comprised patients with on-going substance use and/or homelessness, Group 3 included patients with severe neuropsychiatric disease (i.e. prior suicidal attempts) and/or medical co-morbidities (i.e. history of congestive heart failure). Patients were followed by physicians and pharmacist specialized in HIV where frequency of follow-up and laboratory testing was based on monitoring group assignment (Additional file 1: Table S1 and Additional file 2: Table S2). Pharmacists verified: (1) potential medication interactions; (2) HIV antiretroviral adherence; (3) HCV health education; (4) insurance coverage for access to HCV medication; and (5) patient understanding of HCV medication dosing, administration, and possible side effects. During HCV treatment, HIV pharmacists assist in monitoring: (1) adherence to HCV therapy; (2) critical laboratory results; (3) HCV medication and adjunctive therapy access during treatment course; and (4) unexpected medication interactions/clinical complaints not reported by the patient to physicians.

### Study outcomes

The primary outcome was the difference in the number of referred patients who initiated HCV therapy in both cohorts during the study period. Secondary outcomes were: (1) referral rates by HIV primary care providers for HCV treatment consideration and (2) proportion of patients that following HCV therapy achieved sustained viral response (SVR).

### Independent variables

The following independent variables were assessed: (1) sociodemographic (age, sex, race/ethnicity, HIV risk factor); (2) HIV related (CD4 cell count, HIV viral load and proportion of patients on antiretroviral therapy); (3) HCV viral load and genotype; (4) histological severity of liver disease according to modified Knodell score; (5) neuropsychiatric history, including proportion of patients on any psychotropic medications; (6) history of self-reported illicit substance and/or alcohol use within the last 3 months of HCV treatment evaluation by completion of the ASSIST instrument [13] and subsequent evaluation by the substance abuse counselor and (7) laboratory values [hemoglobin, platelet count, alanine aminotransferase (ALT), international normalized ratio (INR)].

### Statistics

Baseline comparison of clinical characteristics between patients referred to the two clinic models was evaluated using exact Wilcoxon tests for continuous and Fisher’s test for categorical variables. Among patients referred to each clinic model differences in reasons for deferring HCV therapy were explored using chi-square analyses. Bivariate analyses were used to explore the effect of each independent variable in predicting the decision for HCV treatment initiation. Then, we fitted a multiple logistic regression model to adjust for confounding and effect modification of variables associated with HCV therapy initiation using the co-variates found to be significant in prior bivariate analysis (p <0.10). We estimated referral rates as the number of referrals per patient-year of follow-up during the study period. The beginning of follow-up for the hepatology or primary care group was defined as the later of either the date of first date of each cohort study period or the date of first visit to each co-infection clinic model. The end of follow-up for each group was the earlier of either the date of last visit to the co-infection clinic model or the end of the two respective study periods. The referral rate ratio and 95% confidence interval was estimated with its associated p-value. Analyses were performed using NCCSS version 8.0 (Kaysville, Utah, USA).

## Results

**Figure 1:**
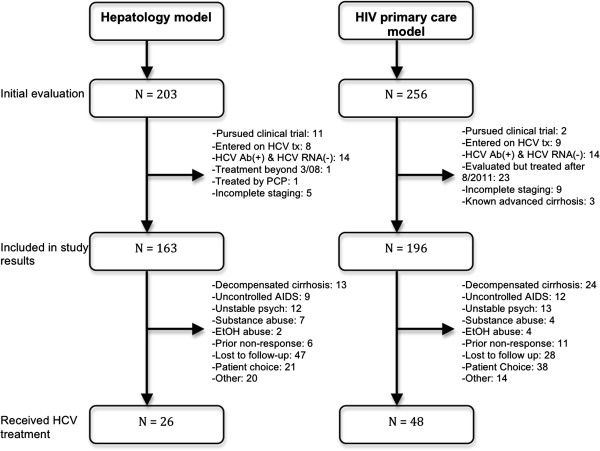
Flow of included patients.

From a total of 667 and 616 HIV/HCV co-infected patients during the studied periods, 256 and 203 were referred for HCV treatment evaluation to the HIV primary care and hepatology models, respectively. The HCV prevalence was similar during studied periods, HIV primary care model (17%) and hepatology (18%). The patient referral rate for HCV treatment did not differ during the 2 study periods, 0.12 vs. 0.10/patient-year for HIV primary care and hepatology models [difference -0.02, 95% confidence intervals (CI) -0.03 to 0.06; p = 0.18]. Main reasons for exclusion of 100 patients after initial visit are shown in Figure 1. The final study cohorts were composed of 196 and 163 patients for the HIV primary care and the hepatology models, respectively. Of note there was no difference in the proportion of patients excluded in both clinic models due to prior HCV treatment null-response (11/196 vs. 6/163; p = 0.39), Figure 1.

Patients referred to the HIV primary care model were older (median 48 vs. 45 years), had a higher proportion of non-hispanics (85 vs. 74%) and reported more frequently heterosexual sex as HIV risk factor (24 vs. 15%) than those referred to the hepatology model. They also had higher median CD4+ cell counts (431 vs. 372/mm^3^) and platelet counts (208 vs. 184 × 103/mm^3^) at the time of HCV treatment consideration (Table 1). There were no differences between clinic model cohorts of referred patients in plasma HIV or HCV viral load, HCV genotype distribution, liver fibrosis score, proportion of patients -receiving HIV therapy, -with neuropsychiatric history, -on psychotropic medications. Among patients referred to the HIV primary care model, 37% reported use of drugs or heavy alcohol within 3 months of HCV treatment consideration (Table 1).

**Table 1:** Characteristics of the patients assessed for hepatitis C treatment consideration according to clinic model

**Clinic model**	**Hepatology**	**HIV primary care**	***P *value**
	**(n = 163)**	**(n = 196)**	
Median age - years (range)	45(26–73)	48(19–75)	0.02^a^
Sex: Male (%)	137(84)	165(84)	0.97^b^
Race			
White	115	138	0.20^c^
Black	39	50	
Asian	0	3	
Other/unknown	9	5	
Ethnicity			
Hispanic	35	26	0.03^c^
Not-Hispanic	121	167	
Unknown	7	3	
HIV risk factors			
Gay/bisexual	79	78	0.003^c^
Heterosexual	24	46	
Intravenous drug use	3	8	
Hemophilia	8	5	
combination	25	47	
Other/unknown	24	12	
Median CD4+ cell count - cells/mm^3^ (range)^1^	372 (29–1179)	431 (38–1612)	0.05^a^
Median HIV load - log_10_ copies/mL (range)	2.6 (2.6–5.8)	2.6 (2.6–6.6)	0.12^a^
Number on HAART therapy - %	132 (81)	172 (88)	0.08^b^
Hepatitis C genotype^2^			
Genotype 1,4	125	158	0.68^c^
Genotype 2,3	31	35	
Median HCV load - log_10_ copies/mL (range)^3^	6.29 (1.9–8.4)	6.43 (2.2–8.4)	0.43^a^
Liver biopsy scores ^4^			
F0-2	39	42	0.95^c^
F3,4	37	39	
Any neuropsychiatry history – %	100 (61%)	119 (61%)	0.85^b^
On any psychiatry medications - %	75 (46%)	88 (45%)	0.83^b^
Drugs or alcohol use within 3months of HCV treatment consideration			
No	71	115	0.0001^c^
yes	45	72	
unknown	47	9	
Median hemoglobin levels - g/dL (range)	14.7 (9–18.7)	14.3 (7.7–17.6)	0.13^a^
Median platelet count -1000/mm^3^ (range)	184 (44–531)	208 (41–548)	0.004^a^
Median ALT - U/L (range)	68 (20–980)	57 (12–1280)	0.51^a^
Median INR (range)	1.1 (0.9–2.3)	1.0 (0.9–2.6)	0.05^a^

ALT = Alanine aminotransferase ; AST = Aspartate aminotransferase; INR = International normalized ratio.

^1^ CD4+ counts available in 150 of 163 patients in the hepatology model and in all patients in HIV primary care model.

^2^ HCV genotype was not available in seven patients in hepatology model and one in HIV primary care model.

^3^ Liver biopsies: 70 in hepatology and 67 in HIV primary care models.

^4^ Two patients in the hepatology model had no available HCV viral load.

^a^ Wilcoxon Rank-Sum Test.

^b^ Chi-square Test.

^c^ Fisher’s exact Test.

While in the HIV primary care model a higher proportion of patients (19 vs. 13%) chose to defer HCV therapy due to personal reasons (e.g. did not want to request time off at work), in the hepatology model more patients failed to initiate HCV therapy due to loss-to-follow up (29 vs. 14%), overall comparison χ^2^ 15.6, df = 8, p = 0.03 (Figure 1).

### Treatment rates and predictors of Hepatitis C treatment

More patients were treated for HCV in the HIV primary care model than in the hepatology model (48 vs. 26, p = 0.043). This difference was more apparent in the second year of implementation of the HIV primary care model, when comparing the proportion of patients treated for HCV by year of study periods: year 1 (9 vs. 13%), year 2 (35 vs. 11%), year 3 (44 vs. 28%), for HIV primary care vs. hepatology models, respectively, χ^2^ 8.1, df = 2, p =0.02.

Patients treated in the HIV primary care model had a higher median CD4+ cell count (522 vs. 375/mm^3^), platelet count (224 vs. 178 × 10^3^/mm^3^, p = 0.01) and frequency of drugs or alcohol use within 3 months of HCV therapy evaluation (33 vs. 19%). Among those treated for HCV, there were no differences between clinic models in the age, race/ethnicity, HIV/HCV viral load, proportion on HIV therapy, HCV genotype distribution, liver fibrosis score, relapse after prior HCV treatment or prevalence of psychiatric disease (Table 2). When comparing clinical characteristics of HCV treated and untreated patients within each model, in the hepatology model there were no clinical variables associated with the decision to initiate HCV treatment. However, patients treated for HCV under the HIV primary care model had higher CD4+ cell counts, lower rates of psychiatric illness, and were prescribed fewer psychotropic medications (Table 3). Using bivariate categorical analysis, independent predictors of HCV therapy initiation included being referred to the HIV primary care model [odds ratio (OR) 1.8, 95% CI 1.1–2.9, p = 0.04]; having a higher CD4+ count (≥400/mm3, OR: 1.8, 95% CI 1.1–3.0) and ALT levels (> 63 U/L, OR: 1.9, 95% CI 1.2–2.9). Prior psychiatric medication use correlated negatively with HCV treatment initiation (OR: 0.6, 95% CI 0.3–0.99). Logistic regression analysis adjusting for all aforementioned significant variables showed that the strongest predictor of HCV treatment initiation was CD4+ count (≥400/mm3, OR: 2.1, 95% CI 1.2–3.6, p = 0.01) whereas a history of prior psychiatric medication use decreased the chances of HCV treatment initiation (OR: 0.5, 95% CI 0.3–0.9, p = 0.02), (Table 4).

**Table 2:** Characteristics of the patients treated for hepatitis C according to clinic model and treatment outcomes

**Clinic model**	**Hepatology**	**HIV primary care**	***P *value**
	**(n = 26)**	**(n = 48)**	
Median age - years (range)	44 (27–62)	49 (19–61)	0.18^a^
Sex: Male (%)	23 (88)	38 (79)	0.32^b^
Race			
White	21	32	0.44^c^
Black	4	13	
Other/unknown	1	3	
Ethnicity			
Hispanic	5	7	0.12^c^
Not-Hispanic	19	41	
Unknown	2	0	
HIV risk factors			
Gay/bisexual	8	21	0.008^c^
Heterosexual	5	14	
Intravenous drug use	1	0	
Hemophilia	3	0	
combination	3	11	
Other/unknown	6	2	
Median CD4+ cell count - cells/mm^3^ (range)	375 (149–1179)	522 (130–1142)	0.02^a^
Number with undetectable HIV load (%)	22 (85)	29 (83)	0.88^b^
Hepatitis C genotype^2^			
Genotype 1/4	19	39	0.42^b^
Genotype 2/3	7	9	
Median HCV load - log_10_ copies/mL (range)	6.04 (5.1–7.7)	6.41 (3.0–7.6)	0.88^a^
Liver biopsy scores ^1^			
F0-2	8	12	0.37^c^
F3,4	9	23	
Number with relapse after prior HCV treatment (%)	5 (19)	5 (11)	0.29^b^
Any neuropsychiatry history – %	17 (65)	23 (48)	0.15^b^
On any psychiatry medications - %	11 (42)	15 (31)	0.34^b^
Drugs or alcohol use within 3months of HCV treatment consideration			
No	12	32	0.0001^c^
Yes	5	16	
Unknown	9	0	
Median hemoglobin levels - g/dL (range)	14.8 (11.1–16.9)	14.5(12.1–16.6)	0.17^a^
Median platelet count -1000/mm^3^ (range)	178 (56–367)	224 (113–418)	0.01^a^
Median ALT - U/L (range)	71 (22–980)	64 (19–1087)	0.48^a^
Median INR (range)	1.1 (0.9–1.4)	1.0 (0.9–1.2)	0.69^a^
**HCV treatment outcomes**			
№ Patients with rapid viral response (%)	4 (15)	12 (25)	0.34^b^
№ Patients with complete early viral response (%)	9 (35)	26 (54)	0.11^b^
№ Patients with end of treatment response (%)	12 (46)	24 (50)	0.75^b^
№ Patients with sustained viral response (%)	9 (35)	21 (44)	0.45^b^
№ Patients who discontinued HCV therapy due to non-viral response (%)	8 (31)	9 (19)	0.24^b^
№ Patients who discontinued HCV therapy due to treatment-related side effects (%)	4 (15)	14 (29)	0.16^b^
№ Patients lost to follow-up (%)	2 (8)	4 (8)	0.92^b^

^1^ Liver biopsies were not performed in 2 and 6 patients with genotype 1 in the hepatology and HIV primary care models respectively.

^a^ Wilcoxon Rank-Sum Test.

^b^ Chi-square Test.

^c^ Fisher’s exact Test.

**Table 3:** Comparison of characteristics of the treated vs. untreated patients for hepatitis C according to clinic model

**Clinic Model**	**Hepatology**			**HIV Primary care**		
	**Treated**	**Not treated**	***P***	**Treated**	**Not treated**	***P***
	**(n = 26)**	**(n = 137)**		**(n = 48)**	**(n = 148)**	
Median age - years (range)	44 (27–62)	45 (26–73)	0.73^a^	49 (19–61)	48 ( 23–75)	0.46^a^
Sex: Male (%)	23 (89)	114 (83)	0.50^b^	38 (79)	127 (86)	0.27^b^
Race						
White	21	94	0.46^c^	32	106	0.63^c^
Black	4	35		13	37	
Other/unknown	1	8		2	5	
Ethnicity						
Hispanic	5	30	0.64^c^	7	19	0.59^c^
Not-Hispanic	19	102		41	16	
Unknown	2	5		0	3	
HIV risk factors						
Gay/bisexual	8	71	0.18^c^	21	57	0.33^c^
Heterosexual	5	19		14	32	
Intravenous drug use	1	2		0	8	
Hemophilia	3	5		0	5	
combination	3	22		11	36	
Other/unknown	6	18		2	10	
Median CD4+ cell count - cells/mm^3^ (range)	375 (149–1179)	371 (29–1025)	0.68^a^	522 (130–1142)	388 (38–1612)	0.005^a^
Number of patients on HIV therapy (%)	22 (85%)	110 (80%)	0.61^b^	44 (92%)	128 (87%)	0.74^b^
Number with undetectable HIV load (%)	22 (85%)	95 (69%)	0.11^b^	40 (83%)	115 (78%)	0.41^b^
Hepatitis C genotype^1^						
Genotype 1/4	19	106	0.32^c^	39	119	0.90^b^
Genotype 2/3	7	24		9	26	
Median HCV load - log_10_ copies/mL (range)^2^	6.0 (5.1–7.7)	6.3 (1.9–8.4)	0.71^a^	6.4 (3.01–7.6)	6.4 (2.2–8.4)	0.19^a^
Liver biopsy scores						
F0-2	8	31	0.10^c^	12	30	0.19^c^
F3-4	9	28		13	26	
unknown	9	78		23	92	
Any neuropsychiatry history – %	17 (65%)	83(61%)	0.65^b^	23(48%)	96(65%)	0.04^b^
On any psychiatry medications - %	11 (42%)	64(47)	0.68^b^	15(31%)	73(49)	0.03^b^
Drugs or alcohol use within 3months of HCV treatment consideration						
No	12	59	0.55^c^	32	83	0.15^c^
Yes	5	40		16	56	
Unknown	9	38		0	9	
Median hemoglobin levels - g/dL (range)	14.8 (11.1–16.9)	14.7 (9–18.7)	0.37^a^	14.5 (12.1–16.6)	14.2 (7.7–17.6)	0.67^a^
Median platelet count -1000/mm^3^ (range)	178 (56–367)	185 (44–531)	0.47^a^	224 (113–418)	207 (41–548)	0.18^a^
Median ALT - U/L (range)	71 (22–980)	67 (20–488)	0.56^a^	64 (19–1087)	56 (12–1280)	0.25^a^
Median INR (range)	1.1 (0.9–1.4)	1.1 (0.9–2.3)	0.33^a^	1.0 (0.9–1.2)	1.0 (0.9–2.6)	0.66^a^

^1^ In the hepatology model (7) and HIV primary care model (3) patients had no available genotype, all in untreated group, there were no patients with genotype #4 in treated groups.

^2^ Two patients in the untreated group in the hepatology model had no available HCV viral load.

^a^ Wilcoxon Rank-Sum Test.

^b^ Chi-square Test.

^c^ Fisher’s exact Test.

**Table 4:** Predictors of Hepatitis C treatment among patients referred to the hepatology (n = 163) and HIV primary care models (n = 196) in unadjusted and adjusted analyses

**Covariate**	**Unadjusted**	***P *value**	**Adjusted**	***P *value**
	**OR (95% CI)**		**OR (95% CI)**	
Hepatitis C treatment				
(HIV primary care vs. hepatology model )	1.7 (1.0–2.9)	0.04	1.6 (0.9–2.8)	0.08
CD4+ cell count ^1^				
(≥400 vs. < 400 cells/mm^3^)	1.8 (1.1–3.03)	0.03	2.1 (1.2–3.6)	0.01
Having detectable HIV load				
(≥400 vs. < 400 copies/mL)	0.5 (0.3–1.1)	0.09	0.6 (0.3–1.1)	0.09
On any psychiatry medications				
(any vs. none)	0.6 (0.3–0.99)	0.04	0.5 (0.3–0.9)	0.02
Alanine aminotransferase levels				
(≥63 vs. < 63U/L)	1.9 (1.2–2.9)	0.002	1.4 (0.8–2.3)	0.3
Any neuropsychiatry history				
(yes vs. no)	0.7 (0.4–1.2)	0.17		
Age - years				
Per year increase	1.0 (0.9–1.03)	0.88		
Sex - biological				
(male vs. female)	1.2 (0.6–2.3)	0.65		
Race				
(white vs. non-white)	1.2 (0.6–2.5)	0.61		
Ethnicity:				
(non-hispanic vs. hispanic)	1.6 (0.2–13.4)	0.68		
HIV risk factors				
Men having sex with men	Reference			
Heterosexual	1.6 (0.9–3.2)	0.14		
Intravenous drug use	0.4 (0.1–3.6)	0.44		
Hemophilia	1.3 (0.3–5.1)	0.68		
Combination	1.1 (0.5–2.1)	0.86		
Hepatitis C genotype ^2^				
(1/4 vs. 2/3)	0.8 (0.4–1.5)	0.50		
Liver biopsy scores ^3^				
(Proportion F3-4 vs. F0-2)	1.2 (0.6–2.5)	0.55		
Drugs or alcohol use within 3 months of				
HCV evaluation^4^				
(yes vs. no)	0.7 (0.4–1.3)	0.24		
HCV load - log_10_ copies/mL^5^				
(>6.33 vs. ≤ 6.33)	0.8 (0.5–1.4)	0.46		
Hemoglobin levels				
(>14.1 vs. ≤ 1 4.1 g/dL)	1.5 (0.8–2.6)	0.13		
Platelet count				
(>139 vs. ≤ 139 - 1000/mm^3^)	1.3 (0.8–2.1)	0.31		
INR levels				
(>1.1 vs. ≤1.1)	0.5 (0.2–1.3)	0.16		

^1^CD4+ counts available in 150 of 163 patients in the hepatology model and in all patients in HIV primary care model.

^2^ HCV genotype was not available in seven patients in hepatology model and one in HIV primary care model.

^3^ Liver biopsies: 70 in hepatology and 67 in HIV primary care models.

^4^ No data available in 47 patients in the hepatology model and in 9 patients in the HIV primary care model.

^5^ Two patients in the hepatology model had no available HCV viral load.

### Outcomes of HCV treatment

Similar proportions of patients achieved undetectable HCV viral load -at week 4 (rapid viral response [RVR]), - at week 12 (complete early viral response [cEVR]) and at week 48 (end of treatment response [ETR]) in both models (HIV primary care vs. hepatology): RVR [12/48(25%) vs. 4/26(15%)], cEVR [26/48(54%) vs. 9/26(35%)] and ETR (24/48(50%, vs. 12/26(46%)]. Twenty-one of 48 patients (44%) in the HIV primary care and 9 of 26 patients (35%) in the hepatology model achieved HCV SVR (p =0.45).

When comparing both models (primary care vs. hepatology), there were no differences in the overall HCV treatment discontinuations rates [27/48(56%) vs. 14/26(54%), p = 0.84] or cause-specific reasons HCV-treatment discontinuation rates: (1) non-viral response [9 /48 (18%) vs. 8/26 (31%), p = 0.24]; (2) HCV therapy-related adverse events [14/48 (29%) vs. 4/26 (16%), p =0.19]; and loss to follow-up (4/ 48 (8%) vs. 2/26 (8%), p = 0.92]. The median time for HCV treatment discontinuation due to adverse events in the HIV primary care model was 12 weeks (range 1–55) and was comparable to the hepatology model (median 8 weeks, range 1–40). The reasons for treatment discontinuation in the hepatology model (n = 4) were hematologic (2) and neuropsychiatric (2) side effects. In the HIV primary care model, main reasons for adverse-events HCV treatment discontinuation (n = 14) were neuropsychiatric (7) and somatic (7) side effects. The somatic side effects included: severe fatigue (3), anorexia /weight loss (2), dizziness (1) and skin rash (1). In the HIV primary care model, 16 (33%) HCV treated patients were actively using illicit substances of whom 2 were using intravenous drugs and 4 were homeless. None of the treated homeless patients were lost to follow-up.

Among patients treated in the HIV primary care model, 17 were considered high-risk: substance use and/or homelessness (n = 10) and severe psychiatric and/or medical comorbidities (n = 7). The group of ‘high-risk’ patients was assigned to special HCV treatment monitoring strategies as described in Additional file 1: Table S1. There were no statistical differences between high-risk vs. non high-risk patients in the rates of SVR, discontinuations due to non-viral response or HCV therapy-related adverse events or loss to follow-up (Table 5). However, the difference in point estimates for loss to follow-up (18% vs. 3% [p = 0.08] for high and non-high risk, respectively) partially validates the criteria we used to identify high risk patients.

**Table 5:** Characteristics and outcomes of patients treated for hepatitis C in the HIV primary care model dichotomized according to risk category based on assigned HCV treatment monitoring group*

**Clinic model**	**High-risk**	**Non-high-risk**	***P *value**
	**(n = 17)**	**(n = 31)**	
Median age - years (range)	49 (19–61)	47 ( 33–61)	0.77^a^
Sex: Male (%)	14 (82)	24 (77)	0.68^b^
Race			
White	13	19	0.14^c^
Black	2	11	
Other/unknown	2	1	
Ethnicity			
Hispanic	2	5	0.68^c^
Not-Hispanic	15	26	
Median T CD4+ cell count - cells/mm^3^ (range)	515(130–1136)	529(170–1140)	0.55^a^
Number with undetectable HIV load (%)	14 (82)	26 (84)	0.91^b^
Hepatitis C genotype			
Genotype 1/4	13	26	0.53^b^
Genotype 2/3	4	5	
Median HCV load - log_10_ copies/mL (range)	6.44(4.05–7.46)	6.40(3.0–7.6)	0.73^a^
Liver biopsy scores ^1^			
F0-2	4	8	0.51^c^
F3,4	6	7	
Median Hemoglobin levels - g/dL (range)	14.5(12.9–15.8)	14.4(12.1–16.6)	0.63^a^
Median Platelet count -1000/mm^3^ (range)	191(113–418)	227(119–402)	0.38^a^
Median ALT - U/L (range)	85(22–301)	56(19–1087)	0.08^a^
**HCV treatment outcomes**			
№ Patients with Sustained viral response (%)	5(29)	16(52)	0.14^b^
№ Patients who discontinued HCV therapy due to non-viral response (%)	2(12)	7(23)	0.36^b^
№ Patients who discontinued HCV therapy due to treatment-related side effects (%)	6(35)	8(26)	0.49^b^
№ Patients lost to follow-up (%)	3(18)	1(3)	0.08^b^

* High-risk patients comprised patients with substance use and/or homelessness (n = 10) and severe psychiatry and/or medical comorbidities (n = 7).

^1^ Liver biopsies were not performed in 6 patients with genotype 1 in the high-risk (n = 3) and non-high risk (n = 3) respectively.

^a^ Wilcoxon Rank-Sum Test.

^b^ Chi-square test.

^c^ Fisher’s exact test.

## Discussion

Our comparison of the performance of an HIV primary care model for the treatment of HCV among HIV-infected patients in comparison to a subspecialty hepatology model allows the following conclusions to be made: (1) In the HIV primary care model there were more patients treated for HCV and fewer were lost to follow-up; (2) HCV treatment referral rates did not differ during two study periods; (3) discontinuation rates were similar in both models despite the increased prevalence of ongoing substance use in the HIV primary care model; (4) the rate of HCV cure (SVR) was similar in both clinic models.

The idea of using an HIV primary care model for the treatment of HCV is not new [8,14-17]. However, a novel component of our approach was the integration of HIV clinical pharmacists to enhance protocol adherence and patient safety. Undoubtedly, the role of the pharmacists is becoming more prominent since the introduction of HCV direct acting antivirals in co-infected individuals, where attention to medication interactions is particularly important [18].

The patient referral rate for HCV treatment did not increase during the HIV primary care model, but referred patients had less obvious clinical or laboratory signs of decompensated liver disease such as thrombocytopenia or coagulopathy (Table 2). It is not clear whether this was the result of the increased awareness among HIV providers about importance of HCV treatment in the HIV primary care model. What is known is that in the HIV primary care model, 33% of patients treated for HCV had history of ongoing substance use and 4 patients were homeless. Despite this ‘high risk population’ treated for HCV by the HIV primary care model, the proportions of HCV treatment discontinuation (either due to adverse events or loss to follow-up) and cure were similar to the hepatology model. Moreover, the treatment outcomes in the high-risk patients were similar to the non-high risk patients treated in the HIV primary care model. In particular there was no difference in the rate of treatment discontinuation due to adverse events (Table 5). We acknowledge that our samples size was small to detect as significant differences between high-risk and non-high risk treated patients. But the message to highlight is that the collaborative monitoring strategy use by the HIV primary care model allowed the HCV cure of patients that may have been rendered unfavorable HCV treatment candidates in other sub-specialty models [2]. The finding that patients with unstable psychiatric conditions, higher HIV viral loads and lower CD4 cell counts were less likely to be treated in the HIV primary care model was in line with the Owen Clinic protocol of working prospectively with patients to link them to care and, once they are more stable and engaged, initiate HCV therapy [19]. This contrasted with the lack of association with any of the studied variables to predict HCV therapy initiation in the hepatology model.

The present study found no significant differences in the rates of HCV SVR and discontinuation of HCV therapy due to adverse events, however, the SVR trend was greater in patients treated on the HIV primary care clinic (44 vs. 35%) despite having almost double discontinuations due to adverse events of patients treated by the hepatology clinic (29 vs. 16%), perhaps due our lack of sample size to detect those differences as significant (e.g. current sample size has 10% power to detect a difference of 9% in SVR as significant). In the absence of a difference in the proportion of patients with viral relapse after prior HCV therapy in both treatment models, we believe that this observation may be explained by unmeasured clinical factors such as: 1) potential better interleukin-28B gene polymorphism allele profile in patients treated in the HIV primary care model [20], since this test was not available in our institution at the time we performed study; 2) a positive effect of HIV primary care model in motivating patients while on HCV therapy that led to improved adherence and hence chances of cure in those who were able to tolerate HCV therapy [21]. This study has important limitations. First, this was not a randomized clinical trial; rather the aim was to compare the performance of 2 clinic-based models with balanced and overall comparable study populations. Second, the HIV primary care model did not treat patients with advanced cirrhosis given that these individuals are more prone to develop severe adverse events. Thus, our findings do not apply to patients with advanced liver disease, who still stand to benefit from specialized care by hepatologists. Third, we cannot rule out that the increasing rates of HCV treatment initiation over time in the HIV primary care model could be the result of secular trends: (a) aging of cohort and more urgency for treatment, (albeit we found no difference in liver fibrosis scores in both cohorts when liver biopsies were performed); (b) concurrent emphasis in the literature of epidemiologic evidence that liver disease is one of the leading causes of death in HIV-infected people, which may have motivated more treatment [22]. It could be argued that HCV treatment uptake rate in the HIV primary care model was not ideal (25%). However, our reported HCV-treatment rates are higher than a recently reported aggressive program to engage HIV/HCV co-infected patients in care sponsored by the United States National Institutes of Health [23] and similar to many European countries,[24] despite the fact we had considerably less accrual time than other clinic-based studies [16]. We believe that our study results underestimate the positive impact of the HIV primary care model in the HCV treatment uptake rate, since 23 patients in this model were excluded from the analysis because: (1) Three patients were still receiving HCV therapy and therefore we could not assess their final outcomes; (2) twenty patients who were staged and eligible for conventional HCV therapy within study period elected to wait for HCV protease inhibitors availability and initiated HCV treatment right before or after 30 July 2011, (Figure 1).

The fact that 10% of patients in both cohorts presented with advanced cirrhosis and were not eligible for HCV therapy highlights the importance of reducing disparities in access to HCV care in the HIV population [3,25]. New HCV therapies offer higher chances of cure, simpler and hopefully less toxic regimens [26,27]. However, to scale up HCV treatment among the HIV infected population, we will need an inclusive collaborative approach that reduces the negative referral bias of physicians when making HCV treatment decisions in vulnerable populations with ongoing barriers to care [22,28,29]. We believe that the HIV primary care model could be useful in other settings and countries burdened by the high prevalence of HCV and difficult to treat urban, poor, marginalized populations that require both more efficacious HCV therapies and newer collaborative models of care such as the one described here [30].

In conclusion, in this exploratory analysis, the use of an HIV primary care model supported by pharmacists specialized in HIV care increased the number of patients who initiate and successfully finish HCV therapy with comparable virological outcomes to a subspecialty hepatology model, highlighting the importance of increasing the absolute number of HIV-infected patients treated for HCV at any given time.

## Competing interests

The authors have no conflict of interest.

## Authors’ contributions

EC Clinical care, IRB request supervision, study design, data collection, data analyses and manuscript preparation. LH, Clinical care, IRB preparation and submission, study design, data collection and data analyses. CB, BC study design, IRB request supervision and clinical care. FT & DW clinical care. Wm CM, study design, IRB submission supervision and data analyses. All authors read and approved the final manuscript.

## Supplementary Material

Additional file 1: Table S1 Clinic visit schedule for HIV patients on HCV treatment based on their specific barriers and/or medical co-morbidities (in weeks). Click here for file

Additional file 2: Table S2 Minimal laboratory monitoring for HIV co-infected patients while on HCV treatment with pegylated Interferon and ribavarin^1^ (time in weeks). Click here for file
