# Supplementary material for: Increasing Hepatitis C treatment uptake among HIV-infected patients using an HIV primary care model
Source: AIDS Res Ther. 2013 Mar 28;10:9. doi: 10.1186/1742-6405-10-9 (PMC3620560; doi:10.1186/1742-6405-10-9)
Supplement: Additional file 1: Table S1 — Clinic visit schedule for HIV patients on HCV treatment based on their specific barriers and/or medical co-morbidities (in weeks). [file 1742-6405-10-9-S1.docx]

**Additional file 1: Table S1**

**Clinic visit schedule for HIV patients on HCV treatment based on their specific barriers and/or medical co-morbidities (in weeks)**

| Homeless^1^ | 0 | 1 | 2 | 3 | 4 | 5 | 6 | 7 | 8 | 9 | 10 | 11 | 12 | 14 | 16 | 18 | 20 | 22 | 24 | 26 | 28 | 30 | 32 | 34 | 36 | 40 | 44 | 48 |
| --- | --- | --- | --- | --- | --- | --- | --- | --- | --- | --- | --- | --- | --- | --- | --- | --- | --- | --- | --- | --- | --- | --- | --- | --- | --- | --- | --- | --- |
| **Group 1** |  |  |  |  |  |  |  |  |  |  |  |  |  |  |  |  |  |  |  |  |  |  |  |  |  |  |  |  |
| Pharmacists | X |  | X |  | X |  |  |  | X |  |  |  | X |  | X |  | X |  | X |  | X |  | X |  | X | X |  | X |
| Providers | X |  |  |  | X |  |  |  | X |  |  |  | X |  | X |  | X |  | X |  | X |  | X |  | X | X | X | X |
| **Group 2** |  |  |  |  |  |  |  |  |  |  |  |  |  |  |  |  |  |  |  |  |  |  |  |  |  |  |  |  |
| Pharmacists | X | X | X | X | X |  | X |  | X |  | X |  | X | X |  | X |  | X |  | X |  | X |  | X |  | X |  | X |
| Providers | X |  |  |  | X |  |  |  | X |  |  |  | X |  | X |  | X |  | X |  | X |  | X |  | X |  | X | X |
| **Group 3** |  |  |  |  |  |  |  |  |  |  |  |  |  |  |  |  |  |  |  |  |  |  |  |  |  |  |  |  |
| Pharmacists | X | X |  | X |  | X |  | X |  | X |  | X |  |  | X | X | X | X | X |  | X |  | X |  | X |  | X | X |
| Providers | X | X | X | X | X |  | X |  | X |  | X |  | X | X | X | X | X | X | X | X | X | X | X | X | X | X | X | X |

**Group 1:** patients without major significant medical comorbidity, social barriers and no ongoing illicit substance use

**Group 2:** patients with ongoing substance use (including intravenous) and/or homelessness^1^

**Group 3:** patients with severe neuropsychiatry disease (including prior suicidal attempts^2^) and/or medical comorbidity

1. Homeless patients receive any parenteral medication required for HCV therapy or its complications in our clinic.

H/o suicidal attempt ^2^

2. Prior suicidal attempt must had occurred at least 24 months prior treatment initiation and recently clear by a formal psychiatry assessment.

**HIV Primary care model team =** Provider **+** Pharmacist
