# Supplementary material for: Increasing Hepatitis C treatment uptake among HIV-infected patients using an HIV primary care model
Source: AIDS Res Ther. 2013 Mar 28;10:9. doi: 10.1186/1742-6405-10-9 (PMC3620560; doi:10.1186/1742-6405-10-9)
Supplement: Additional file 2: Table S2 — Minimal laboratory monitoring for HIV co-infected patients while on HCV treatment with pegylated Interferon and ribavarin1 (time in weeks). [file 1742-6405-10-9-S2.docx]

**Additional file 2: Table S2**

**Minimal laboratory monitoring for HIV co-infected patients while on HCV treatment with pegylated Interferon and ribavarin^1^ (time in weeks).**

|  | | Baseline^2^ | 2 | 4 | 8 | 12 | 16 | 20 | 24 | 28 | 32 | 36 | 40 | 44 | 48 | 52 | 56 | 60 | 64 | 68 | 72 |
| --- | --- | --- | --- | --- | --- | --- | --- | --- | --- | --- | --- | --- | --- | --- | --- | --- | --- | --- | --- | --- | --- |
| CBC | | X | X | X |  | X | X | X | X | X | X | X | X | X | X | X |  | X |  |  | X |
| CMP | | X |  | X |  | X |  |  | X |  |  | X |  |  | X |  |  |  |  |  | X |
| Urinalysis | | X |  |  |  |  |  |  | X |  |  |  |  |  | X |  |  |  |  |  |  |
| HCV RNA | | X |  | X |  | X |  |  | X |  |  | X |  |  | X | X |  | X |  |  | X |
| CD4 | X | |  |  |  | X |  |  | X |  |  | X |  |  | X |  |  |  |  |  |  |
| HIV RNA | | X |  |  |  |  |  |  | X |  |  |  |  |  | X |  |  |  |  |  |  |
| TSH | | X |  |  |  |  |  |  | X |  |  |  |  |  | X |  |  |  |  |  |  |
| STI^3^ | | X |  |  |  | X |  |  | X |  |  | X |  |  | X |  |  |  |  |  | X |
| U-tox | | X |  | X |  | X |  |  | X |  |  | X |  |  | X |  |  |  |  |  | X |
| Lactic acid | | X |  |  |  | X |  |  |  |  |  |  |  |  | X |  |  |  |  |  |  |

CBC= Complete blood cell count, CMP=comprehensive metabolic panel, HCV RNA= Hepatitis C viral load, TSH= Thyroid-stimulating hormone, STI= Sexual transmitted Infection; U-tox= Urine drug screening test.

1. Additional complementary blood test ordered based on patient’s symptoms and/or severity of concurrent medical co-morbidity.

2. Baseline: Week ‘0’ or within 4 weeks of treatment initiation

3. Asymptomatic STI screening includes a rapid plasma reagin test and urine Gonorrhea/Chlamydia polymerase chain reaction

To the right of vertical ‘line’ (weeks 52-72) depicts post-HCV therapy monitoring
